# Long-Interval Prostaglandin F_2α_ Combined with GnRH Improves the Estrus Synchronization and Reproductive Performance of Sheep During the Breeding Season

**DOI:** 10.3390/ani15030336

**Published:** 2025-01-24

**Authors:** Zengyi Duan, Menghao Liu, Junjin Li, Jian Hou

**Affiliations:** State Key Laboratory of Animal Biotech Breeding, College of Biological Sciences, China Agricultural University, Yuan-Ming-Yuan West Road, Haidian District, Beijing 100193, China; zengyiduan@163.com (Z.D.); liumenghao@cau.edu.cn (M.L.); liyuhailan@126.com (J.L.)

**Keywords:** estrus synchronization, prostaglandin F_2α_, GnRH, sheep

## Abstract

To improve the efficiency of prostaglandin F_2α_ (PG)-based estrus synchronization in sheep, this study assessed the effect of the gonadotropin-releasing hormone (GnRH) included in the long-interval PG treatment regimen for sheep estrus synchronization during the breeding season. In experiment 1, 30 multiparous Mongolian sheep (3–4.5 years old) were randomly divided into three groups. In the progesterone (P4)–equine chorionic gonadotropin (eCG) group (P4-eCG, *n* = 10), the ewes were synchronized with intravaginal P4 sponges for 14 days, and received an injection of 330 IU of eCG at sponge withdrawal. In the PG group (*n* = 10), the ewes received two doses of 0.1 mg PG with a 14 day interval. In the PG-GnRH-PG group (*n* = 10), the ewes were synchronized by two doses of 0.1 mg PG with a 14 day interval like that in the PG group, but received 50 μg of GnRH 7 days after the first injection of PG. It was found that, at the end of treatment, the number of corpus luteum on the ovaries and the concentration of the serum P4 in the PG-GnRH-PG group were significantly higher than that of the PG and P4-eCG groups. In experiment 2, 59 multiparous Mongolian sheep (3–4.5 years old) were assigned to three groups, like in experiment 1 (*n* = 20, 20 and 19 for the P4-eCG, PG and PG-GnRH-PG groups, respectively). The estrus of ewes in the PG-GnRH-PG group was more synchronous compared to the PG group. After insemination of the estrus ewes, the pregnancy rate was numerically but not significantly higher in the PG-GnRH-PG group than that in the P4-eCG and the PG groups. In a field test, 285 multiparous Hu sheep (3–4.5 years old) were randomly assigned to a P4-eCG group (*n* = 142) and PG-GnRH-PG group (*n* = 143). Timed artificial insemination showed no significant differences in the rates of pregnancy and lambing between the PG-GnRH-PG and P4-eCG groups. We conclude that the addition of GnRH in the long-interval PG protocol may improve the efficiency of PG-based estrus synchronization, and would represent a potential alternative to the conventional P4-eCG based protocol during the breeding season in sheep.

## Simple Summary

Estrus synchronization is an important animal reproductive technology, but protocols still need to be refined to enhance its practical use in livestock production. The objective of this study was to improve the efficiency of prostaglandin F_2α_-based estrus synchronization in sheep. We found that addition of an injection of gonadotropin-releasing hormone between the two injections of prostaglandin F_2α_ could enhance estrus synchrony and improve the reproductive performance of synchronized ewes, even achieving a comparable efficiency to the conventional progesterone-based method. This modified protocol could provide a promising strategy for estrus synchronization and timed artificial insemination in sheep as well as other domestic animal species.

## 1. Introduction

Estrus synchronization is an important animal reproductive technology, which is frequently used in artificial insemination (AI), superovulation and embryo transfer programs. The most commonly used estrus synchronization protocol for sheep is the insertion of progesterone (P4)-impregnated intravaginal devices into ewes for 12–14 days, followed by the intramuscular injection of equine chorionic gonadotropin (eCG) at device removal (P4-eCG) [1]. However, the P4-eCG-based protocol has been associated with alterations in oocyte quality, which leads to low fertilization rates and impaired embryo development [2,3]. Furthermore, the repeated use of eCG may cause a humoral immune response in ewes and the development of follicular cysts [4]. Moreover, the P4-eCG-based protocol has a potential for environmental contamination because of residual P4 in used devices and the addition of antibiotic agents to avoid vaginitis [2,5]. In contrast, because of rapid metabolization, easiness of application, better animal welfare and the low cost of the utilization of prostaglandin F_2α_ (PG), the PG-based strategy represents an alternative to P4-eCG based protocols for reproductive management in sheep [1,6,7].

Since the discovery of the luteolytic action of PG in 1970 [8], its synthetic analogs have been widely used in estrus synchronization. The conventional PG-based protocol consists of two PG injections administered 9–14 days apart [6], but estrus is expressed over a wide 4-day window, which limits the practical use of this protocol for timed artificial insemination (TAI) in sheep [9]. A high synchronization of estrus of treated ewes was observed when the injection interval was shortened to 7 days (Synchrovine protocol) [10,11]. However, this protocol yielded a lower pregnancy rate than the classical P4-eCG protocol [12]. The poor reproductive performance in the short PG protocol was suggested to be associated with insufficient P4 concentrations in the synchronized ewes, which in turn led to reduced ovulation, conception, prolificacy, and fecundity [13]. Several studies have attempted to improve the reproductive outcome of this program, but have not been successful [7,14,15,16,17].

Extending the interval between the two PG injections would prolong the duration for which pre-ovulatory follicles were exposed to luteal P4 levels [6]. Indeed, two administrations of PG injections 14–16 d apart (long interval) resulted in an enhanced reproductive outcome after TAI [18,19], with a pregnancy rate comparable to that in the P4-eCG based protocol [20,21]. However, the long PG protocol usually results in lower estrous synchrony when compared to the shorter protocol [18,22].

Gonadotropin-releasing hormone (GnRH) is widely used to induce ovulation, promote the formation of accessory corpus luteum (CL) and improve reproductive performance in sheep and cows [23,24,25,26,27,28,29]. In this study, we hypothesized that an injection of GnRH on the 7th day after the first injection of PG in the long-interval PG program (14 days apart) can cause follicular ovulation. On the one hand, the newly formed CL after ovulation can increase the concentrations of P4 during follicular development. On the other hand, a new wave of developing follicles would appear after ovulation, and the synchronization of follicular development would thus be enhanced. After the second injection of PG, the estrus should be more synchronized. We assumed that the long-interval PG-GnRH-PG protocol would result in a better reproductive performance than the long interval PG protocol. Therefore, this study was designed to address the possibility that the addition of GnRH in the long-interval PG-based protocol would improve the efficiency of PG-based estrus synchronization in sheep.

## 2. Materials and Methods

### 2.1. Ethics Approval

The procedure of all animal experiments was in accordance with the animal care policies of China Agricultural University and was approved by the Animal Ethics Committee at the university (AW20701202-3-3).

### 2.2. Location and Animal Management

The experiments were carried out in Balin Right Banner (118°15′ E, 43°12′ N), Chifeng, Inner Mongolia, China, where the average temperature is around 13.3 °C in autumn and −10.5 °C in winter, the breeding seasons of sheep. All ewes were housed in a semi-open yard and fed corn silage and alfalfa hay twice daily (07:00 and 17:00). Mineral salt and fresh water were offered ad libitum.

### 2.3. Experimental Design

#### 2.3.1. Experiment 1

**Figure 1:**
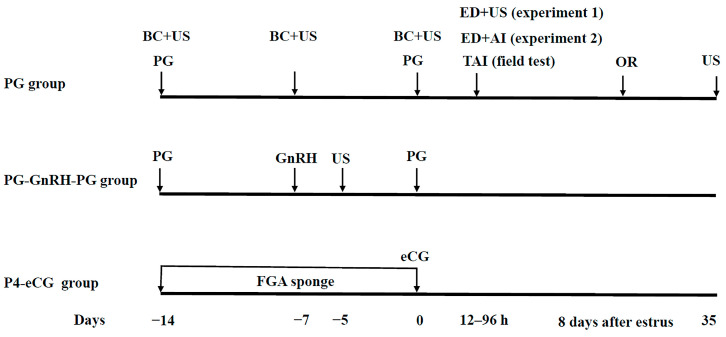
Schematic representation of the experimental design. PG group: two doses of PG (0.1 mg) with 14-day interval. PG-GnRH-PG group: two doses of PG (0.1 mg) with 14-day interval + 50 μg GnRH on Day-7. P4-eCG group: FGA (45 mg) sponge for 14 days + 330 IU eCG on Day 0. BC: blood collection (on Day-14, -7 and 0; Experiment 1). US: ovary assessed by transrectal ultrasonography. ED: estrus detection. ED + US: time of ovulation was assessed every 12 h after estrus detection until the disappearance of the largest follicle by ovarian trans–rectal ultrasonography (experiment 1). AI: artificial insemination. ED + AI: the estrous ewes were subjected to AI 12 and 24 h after estrus (experiment 2). TAI: timed artificial insemination was performed 48 and 60 h after sponge withdrawal (P4-eCG group) or second injection of PG (PG-GnRH-PG group) (field test). OR: ovulation rate was evaluated 8 days after estrus detection by trans-rectal ultrasonography (experiment 1 and 2). US (35): pregnancy was evaluated approximately 35 days after AI by transrectal ultrasonography (experiment 2 and field test).

The aim of this experiment was to determine the ovarian response, estrus response and serum P4 concentrations. During the breeding season (October 2021, autumn), 30 multiparous and non-lactating Mongolian sheep (3–4.5 years old) were randomly allocated to three groups according to their body weight (BW) and body condition score (BCS, scale: 0–5) [30]. In the P4-eCG group (*n* = 10, BW = 49.4 ± 2.9 kg, BCS = 3.2 ± 0.2), the ewes were synchronized with intravaginal polyurethane sponges impregnated with 45 mg fluorogestone acetate (FGA, Beijing Bevic Co., Ltd., Beijing, China) for 14 days, and each ewe received an i.m. injection of 330 IU of eCG (Ningbo Sansheng Pharmaceutical, Ningbo, China) at sponge withdrawal. In the PG group (*n* = 10, BW = 47.5 ± 3.6, BCS = 3.1 ± 0.1), the ewes were intramuscularly injected twice with 0.1 mg PG (Cloprostenol Sodium, Ningbo Sansheng Pharmaceutical) at 14-day intervals. In the PG-GnRH-PG group (*n* = 10, BW = 48.7 ± 2.3, BCS = 3.1 ± 0.3), the ewes were intramuscularly injected twice with 0.1 mg PG at an interval of 14 days, and 50 μg GnRH (Gonadorelin, Ningbo Sansheng Pharmaceutical) was intramuscularly injected 7 days after the first injection of PG. The estrous cyclicity was confirmed by testing CL with ultrasonography in all animals 1 week before starting treatment. All groups of ewes were synchronized starting at a random stage of the estrus cycle. The day of sponge withdrawal (P4-eCG group) or second injection of PG (PG and PG-GnRH-PG group) was defined as Day 0. The estrus signs were evaluated using adult rams wearing a vest; monitoring for estrus started 12 h after sponge withdrawal (P4-eCG group) or second injection of PG (PG and PG-GnRH-PG group) and was performed every 12 h thereafter. Ewes were considered to be in estrus when allowed themselves to be mounted. The estrus ewes were not mated or inseminated, but only for assessment of estrus and ovulation in this experiment (Figure 1).

#### 2.3.2. Experiment 2

This experiment was conducted with the objective of determining estrus synchrony, ovulation rate (OR) and reproductive performance. During the breeding season (November 2021, early winter), a total of 59 multiparous and non-lactating Mongolian sheep (3–4.5 years old) were randomly allocated to three groups as described in experiment 1, including a P4-eCG group (*n* = 20, BW = 52.9 ± 2.1 kg, BCS = 3.3 ± 0.1), PG group (*n* = 20, BW = 49.5 ± 3.1, BCS = 3.1 ± 0.2) and PG-GnRH-PG group (*n* = 19, BW = 50.2 ± 1.9, BCS = 3.1 ± 0.2). Twelve hours post sponge withdrawal (P4-eCG group) or second injection of PG (PG and PG-GnRH-PG group), the estrus signs were detected twice a day (6:00 am and 18:00 pm) for 4 d (96 h) with rams wearing a vest. The estrous ewes were artificially inseminated with fresh semen 12 and 24 h post estrus (Figure 1).

#### 2.3.3. Field Test

The results of experiment 1 and 2 showed that the PG-GnRH-PG protocol produced better estrus synchrony and reproductive performance of the ewes than the PG protocol. To further determine the feasibility of the PG-GnRH-PG protocol, a field test was designed to compare the efficiency between the PG-GnRH-PG and P4-eCG protocols that were used for TAI in a popular breed of Hu sheep. A total of 285 multiparous and non-lactating Hu sheep (3–4.5 years old) were selected and randomly assigned to the P4-eCG group (*n* = 142, BW = 48.5 ± 3.5, BCS = 3.1 ± 0.2) and PG-GnRH-PG group (*n* = 143, BW = 50.3 ± 2.1, BCS = 3.2 ± 0.1) during the breeding season (October 2022, autumn). The estrus synchronization protocols were the same as those in experiment 1. TAI was performed 48 and 60 h post sponge withdrawal (P4-eCG group) or second injection of PG (PG-GnRH-PG group) (Figure 1).

### 2.4. Ultrasonography

During the experiments, transrectal ovarian ultrasonography was performed using a B-mode ultrasound scanner equipped with a 7.5 MHz linear array transducer (HS1600, HONDA, Tokyo, Japan). The transducer was attached to a PVC tube for ease of ultrasound operation. The ultrasonography detection was performed by the same person. In experiment 1, the number, diameter and relative position of all ovarian follicles with a diameter of ≥3 mm and CL on both ovaries were recorded. Time of ovulation was assessed every 12 h after estrus detection until the disappearance of the largest follicle. Ovulation was also determined through ultrasonography to identify if a new CL was present 8 days after estrus detection. In experiment 2, OR (number of corpus luteum/number of ovulated ewes) was evaluated 8 days after estrus detection in ewes showing the estrus behavior after sponge withdrawal or the second PG injection. The pregnancy rate (number of pregnant ewes/number of total ewes × 100) was evaluated approximately 35 days after AI by transrectal ultrasonography, using a 7.5 MHz linear-array transducer (HS1600, HONDA, Japan).

### 2.5. Blood Sample Collection and Assays

For analysis of serum P4 concentrations, blood samples from the jugular vein were collected from all ewes in experiment 1 (on Day-14, -7 and 0). Blood samples were centrifuged (1400× *g*, 15 min at 4 °C) and serum was collected and stored at −20 °C until analysis. The P4 concentrations were determined using a commercial solid phase radioimmunoassay (RIA) kit (Beijing North Institute of Biological Technology, Beijing, China), according to the manufacturer’s instructions. The intra-assay coefficient of variations for P4 was 4.1%, and the inter-assay coefficient was 8.6%. The minimum sensitivity for P4 was 0.05 ng/mL.

### 2.6. Artificial Insemination

Fresh semen was collected with an artificial vagina and assessed for total mobility of the spermatozoa (>80%) before its use. Semen was diluted with skimmed milk at 1:1 volume. Cervical AI was performed using a speculum equipped with a light source and an insemination device (Beijing Bevic Co., Ltd.). The insemination dose per ewe was 0.2 mL containing 200 × 10^6^ spermatozoa, slowly released as deep as possible into the cervix. Both semen collection and AI procedures were performed by experienced personnel.

### 2.7. Statistical Analyses

All data were analyzed with SPSS software (Version: 26.0; IBM, Chicago, IL, USA). Follicular, luteal and hormonal data were analyzed using repeated measure analysis of variance, and post hoc comparisons were carried out using Bonferroni post-tests. The interval from sponge removal/second PG injection to estrus onset, estrus duration, the interval from estrus onset to ovulation or from sponge removal/second PG injection to ovulation, diameters of follicles, ovulation rate and litter size were compared among treatment groups by one-way ANOVA, followed by Duncan’s multiple range test. Estrus rate, occurrence of ovulation, pregnancy and lambing rates were compared using the Fisher exact test or Chi-square test, depending on the number of observations. Continuous variables are presented as the Mean ± SEM, and categorical variables are presented as percentage. Statistical significance was defined as *p* < 0.05.

## 3. Results

### 3.1. Ovarian Response, Serum P4 Concentrations and Estrus Detection in the Synchronized Ewes

**Figure 2:**
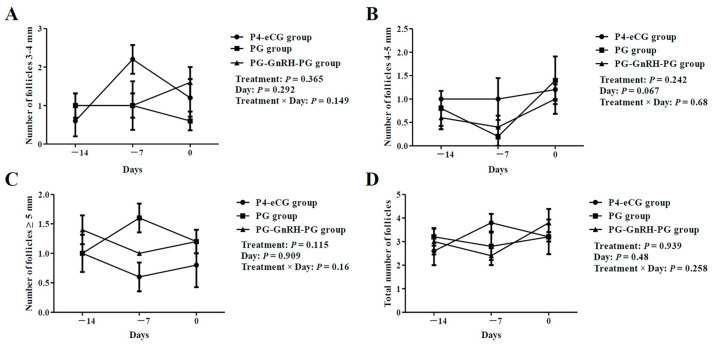
The effect of three synchronization protocols on follicular counts. (**A**) Number of 3–4 mm follicles. (**B**) Number of 4–5 mm follicles. (**C**) Number of large (≥5 mm) follicles. (**D**) Total number of follicles (≥3 mm). P4-eCG group: FGA (45 mg) sponge for 14 days + 330 IU eCG on Day 0. PG group: two doses of PG (0.1 mg) with 14-day interval. PG-GnRH-PG group: two doses of PG (0.1 mg) with 14-day interval + 50 μg GnRH on Day-7. Data are presented as Mean ± SEM. There were no significant differences among experimental groups (*p* > 0.05).

**Figure 3:**
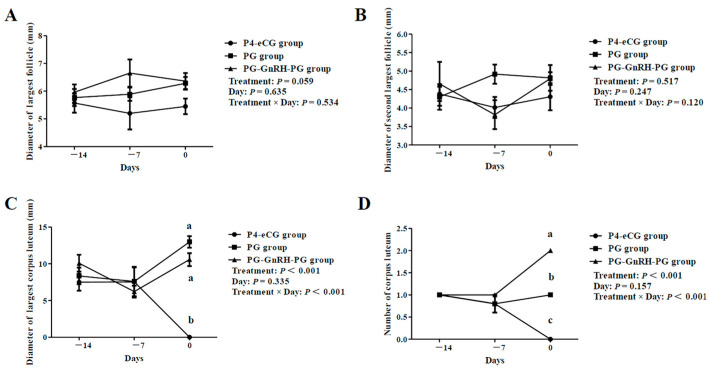
The effect of three synchronization protocols on follicle diameter, CL diameter and CL number. (**A**) Diameter of the largest follicle. (**B**) Diameter of the second largest follicle. (**C**) Diameter of the largest CL. (**D**) Total number of CL. P4-eCG group: FGA (45 mg) sponge for 14 days + 330 IU eCG on Day 0. PG group: two doses of PG (0.1 mg) with 14-day interval. PG-GnRH-PG group: two doses of PG (0.1 mg) with 14-day interval + 50 μg GnRH on Day-7. Data are presented as Mean ± SEM. a, b, and c indicate that the difference among groups is significant at the same time point (*p* < 0.05).

In experiment 1, all three groups of ewes underwent transrectal ovarian ultrasonography to monitor the dynamic of their follicular development during treatment for estrus synchronization. As shown in Figure 2, no significant differences in the numbers of 3–4 mm, 4–5 mm, large (≥5 mm) follicles and the total number of follicles (≥3 mm) were observed among the three groups (*p* > 0.05). There was also no significant difference in the diameters of the largest follicle and the second largest follicle on the ovaries among the three groups (*p* > 0.05; Figure 3A,B).

On Day 14 and Day 7, there was no significant difference in the maximum CL diameter among the three groups (*p* > 0.05). However, on Day 0, the maximum CL diameter in the PG-GnRH-PG and PG groups was significantly larger than that in the P4-eCG group (*p* < 0.05; Figure 3C), and no significant difference in the maximum CL diameter was observed between the PG-GnRH-PG and PG groups. On Day 14 and Day 7, the number of CL on the ovaries was not significantly different among the three groups (*p* > 0.05). However, on Day 0, the CL number in the PG-GnRH-PG group was significantly higher compared to that in the PG and the P4-eCG groups (*p* < 0.05), and the PG group also possessed a higher number of CL than the P4-eCG group (*p* < 0.05; Figure 3D).

**Figure 4:**
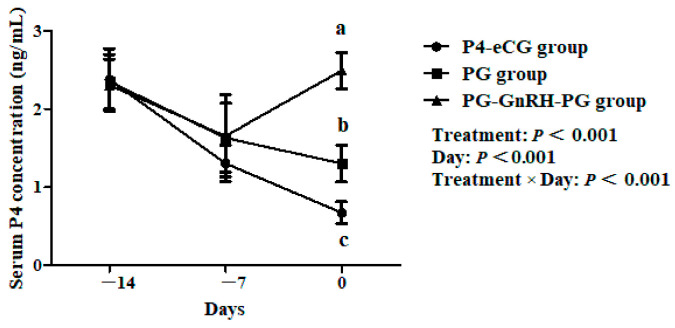
The effect of three synchronization protocols on serum P4 concentrations. P4-eCG group: FGA (45 mg) sponge for 14 days + 330 IU eCG on Day 0. PG group: two doses of PG (0.1 mg) with 14-day interval. PG-GnRH-PG group: two doses of PG (0.1 mg) with 14-day interval + 50 μg GnRH on Day 7. Data are presented as Mean ± SEM. a, b, and c indicate that the difference among groups is significant at the same time point (*p* < 0.05).

On Day 14 and Day 7, there was no significant difference in serum P4 concentrations among the three treatment groups (*p* > 0.05), but on Day 0, the serum P4 concentration was significantly higher in the PG-GnRH-PG and PG groups than in the P4-eCG group (*p* < 0.05), and it was higher in the PG-GnRH-PG group compared to the PG group (*p* < 0.05; Figure 4).

Estrus detection showed no significant difference in the estrus rate among the three groups (*p* > 0.05). Ewes in all groups showed estrus behavior and ovulation. Compared to the P4-eCG group, the interval from sponge removal/second PG injection to estrus onset in the PG and PG-GnRH-PG groups (50.4 h and 52.4 h) was significantly longer than that in the P4-eCG group (44.0 h; *p* < 0.05). No significant difference in such interval was seen between the PG group and PG-GnRH-PG group (*p* > 0.05). The interval from sponge removal/second PG injection to ovulation was significantly longer in the PG group and PG-GnRH-PG group (71.14 h and 73.10 h) compared to the P4-eCG group (63.60 h; *p* < 0.05), with no significant difference between the PG and PG-GnRH-PG groups (*p* > 0.05). There was no significant difference in estrus duration and interval from estrus onset to ovulation among the three groups (*p* > 0.05). The diameters of the largest and second follicles at the estrus onset and large preovulatory follicles were not significantly different among the three groups (*p* > 0.05; Table 1).

### 3.2. Evaluation of Estrus Synchronization and Artificial Insemination of Estrus Ewes

**Figure 5:**
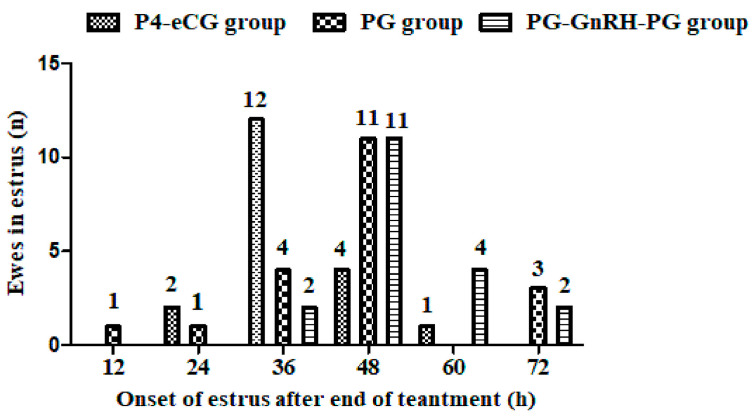
The effect of three synchronization protocols on estrus synchrony. P4-eCG group: FGA (45 mg) sponge for 14 days + 330 IU eCG on Day 0. PG group: two doses of PG (0.1 mg) with 14-day interval. PG-GnRH-PG group: two doses of PG (0.1 mg) with a 14-day interval + 50 μg GnRH on Day-7.

In experiment 2, the synchronization of estrus was evaluated in more detail. As shown in Figure 5, estrus of ewes in the P4-eCG group occurred 24–60 h after removal of the sponge, with a surge in estrus at 36 h (60.0%, 12/20), while estrus in the PG group appeared 12–72 h after the second injection of PG, with the highest number of ewes showing estrus at 48 h (55.0%, 11/20). In the PG-GnRH-PG group, estrus occurred 36–72 h after the second PG injection, and most estrus ewes were seen at 48 h (57.8%, 11/19), indicating that estrus was more synchronous in the PG-GnRH-PG group compared to the PG group (Figure 5).

There was no significant difference in the estrus rate among the three groups (*p* > 0.05). Except for one ewe in the P4-eCG group that did not have estrus, all ewes in the three groups showed estrus behavior. All estrous ewes ovulated except one in the P4-eCG group. Compared to the P4-eCG group, the OR was lower in the PG and PG-GnRH-PG groups (*p* < 0.05), and there was no significant difference in OR between the PG and PG-GnRH-PG groups (*p* > 0.05).

The pregnancy rate of ewes after AI was not significantly different among the three groups (*p* > 0.05), but the PG-GnRH-PG group showed a tendency of increasing pregnancy rate when compared to the P4-eCG and the PG groups, and the pregnancy rate in the PG group was the lowest. No significant difference in the lambing rate was observed among the three groups (*p* > 0.05), with the PG-GnRH-PG group showing a higher lambing rate compared to the P4-eCG and PG groups. The litter size was highest in the P4-eCG group and was lowest in the PG group, but it was not significantly different among the three groups (*p* > 0.05; Table 2).

### 3.3. Field Test for Timed Artificial Insemination

A field test was designed to evaluate the efficiency of PG-GnRH-PG protocol in TAI condition on a larger scale. As shown in Table 3, there were no significant differences in pregnancy and lambing between the PG-GnRH-PG and the P4-eCG groups (*p* > 0.05), and the PG-GnRH-PG group even had higher rates of pregnancy and lambing compared to the P4-eCG group (pregnancy rate 69.9% vs. 63.4%, lambing rate 66.4% vs. 59.2%, *p* > 0.05).

## 4. Discussion

In this study, we hypothesized that an injection of GnRH on the 7th day after the first injection of PG in the long-interval PG program (14 days apart) can cause follicular ovulation. After ovulation a new wave of follicular development would begin, and the estrus and ovulation of ewes would be more synchronized after the second PG injection. In addition, the ovulated follicle induced by GnRH can form a new CL that can increase the concentrations of P4, which would be beneficial to the oocyte development. The results obtained in this study support this hypothesis to some extent.

In an estrus cycle in sheep, a follicular wave emergence occurs approximately every 5 days [31] and the ovine CL is sensitive to PG as early as 3 days after ovulation [10]. Based on this theory, the Synchrovine protocol was developed, where a second PG injection was administrated 7 days after the first PG injection to induce luteolysis of newly formed CL and the largest follicle in wave 1 ovulated soon after [9]. However, the reproductive performance of the Synchrovine protocol was often lower than that of conventional P4-eCG based protocol [12]. Such a low reproductive performance was probably due to the low P4 concentrations during follicular development, as the CL function was terminated prematurely in the Synchrovine protocol [13]. Although prolonging the interval between injections of PG can increase the concentrations of P4 during follicular development and improve the reproductive performance, but the synchronization of estrus was poor [18,19]. In the present study, we administrated a GnRH injection on the 7th day after the first PG injection. We found that at the end of estrus synchronization (Day 0), the number of CL on the ovaries in the PG-GnRH-PG group was significantly higher than that in the PG group and the P4-eCG group. This observation suggested that the injection of GnRH probably caused ovulation, resulting in an increase in the number of CL on the ovary.

Interestingly, we observed that the CL number and the maximum CL diameter were smaller in the P4-eCG group compared to the PG-GnRH-PG and the PG groups. This was probably because the CL in the P4-eCG group underwent degradation during the treatment period, and the CL was completely degraded when the sponge was removed. We found that at the time of GnRH injection, all maximum follicle diameters on the ovaries were above 5 mm. In sheep, the diameter of the largest follicle was 6.4 ± 0.5 mm at the second injection of PG in the Synchrovine protocol [32]. In goats, when using the Synchrovine protocol, the maximum follicle diameter at the second PG injection was 5.0 ± 1.8 mm [33]. The observations in this study further demonstrate that, 7 days after the first injection of PG, the largest follicle on the ovary acquired the ability to ovulate in response to GnRH [24].

During the treatment period, we did not observe significant differences in the numbers of follicles among three groups of ewes. It was suggested that the P4 levels in circulation are correlated with follicular growth [9]. The treatment of ewes with P4 impregnated vaginal sponge resulted in a higher diameter of the largest follicle, and a lower number of small and total follicles when compared to the use of PG protocol [2]. The ewes treated with the PG protocol had a larger ovulatory follicle than the ewes in spontaneous estrus [13]. Considering the difference in action between P4 and PG-based protocols, we surmised that the follicular development of ewes may be different among the three protocols. However, we observed that the follicular development dynamics were similar among the three groups during treatment. More studies are needed to further confirm this in the future.

There was no significant difference in the estrus rate among the three groups, with all groups reaching more than 90% of estrus. Previous studies also showed that the estrus rate in the P4-eCG protocol and PG-based protocol was similar [34]. However, after the end of the treatment, the beginning of estrus and ovulation in the P4-eCG group were significantly earlier than in the PG and the PG-GnRH-PG groups, which is consistent with a previous report [34]. The OR of P4-eCG group was also significantly higher than that in the PG and PG-GnRH-PG groups. Previous studies reported a higher OR in the P4-eCG based protocol than in the PG-based protocol [20,21]. This may be because the ewes in the P4-eCG group were injected with eCG when the sponge was removed. eCG has dual activities of FSH and LH, which can promote follicular development and ovulation. Therefore, the development speed and maturity level of follicles were higher in the P4-eCG group, which promoted faster estrus and more ovulation of ewes.

The estrus behavior of ewes in all treatment groups was mainly synchronized within 72 h after the withdrawal of the sponge or after the second injection of PG. The estrus in the P4-eCG group occurred within 24–60 h, with a surge at 36 h. In the PG and PG-GnRH-PG groups, most ewes (more 50%) began estrus 48 h after the second PG injection, 12 h later than in the P4-eCG group. This was because the injection of eCG in the P4-eCG group induced faster follicle development and an earlier onset of estrus. Compared to the PG group, the window of estrus in the PG-GnRH-PG group was narrowed to within 36 h (36–72 h post second PG injection), which suggested that injection of GnRH had enhanced the estrus synchrony.

In this study, AI after estrus detection resulted in a pregnancy rate of 70.0% in the P4-eCG group, 60.0% in the PG group, and 84.2% in the PG-GnRH-PG group, respectively. Although there were no statistical differences among the three groups in terms of pregnancy and lambing rates due to the small sample size, the PG-GnRH-PG group had the highest pregnancy rate, which to some extent supported our hypothesis that the injection of GnRH could increase reproductive performance. This positive result prompted us to evaluate the efficiency of the protocol in practice in a large sample of ewes. The field test in a popular Hu sheep breed showed that, when used for TAI, the PG-GnRH-PG protocol yielded similar pregnancy and lambing rates to the P4-eCG protocol. Different from Mongolian sheep, Hu sheep are known for precocious puberty, perennial estrus and high fecundity [35]. Our results in these two sheep breeds (Mongolian sheep and Hu sheep) suggest that the PG-GnRH-PG method has a potential to replace the conventional P4-eCG method.

We observed that, at the second injection of PG, the serum P4 concentration in the PG-GnRH-PG group was significantly higher than that in the PG group. The increase in P4 concentrations may be due to two reasons. One is that the injection of GnRH induced ovulation and increased the number of CL on the ovaries. Another is that the GnRH injection caused an increase in LH secretion, which promoted the development and the maintenance of already existing CL on ovaries [36,37]. Many studies have confirmed that P4 supplementation could improve the oocyte and embryo development potential and increase reproductive performance. Supplementation of P4 during superovulation in sheep increased the oocyte fertilization and embryo quality [38,39]. The addition of exogenous P4 during the in vitro maturation of oocytes improved the developmental competence of partially cumulus-denuded bovine oocytes [40]. P4 administration during estrus synchronization with two PG injections in dairy cows increases the pregnancy rate [41]. Therefore, the improvement in the reproductive performance of PG-GnRH-PG group may be attributed to the high P4 concentrations during follicular development.

## 5. Conclusions

In conclusion, in the long-interval PG protocol (14 days apart), an injection of GnRH 7 days after the first injection of PG can increase the number of CL on the ovaries and serum P4 concentrations in ewes, enhance estrus synchrony and improve reproductive performance. This protocol, which we termed PG-GnRH-PG (or PGP for short), represents a modification of the PG-based protocol, and could be promising for estrus synchronization in sheep as well as other ruminant animals. Although the inclusion of GnRH increased some costs when compared to the traditional PG protocol, it improved the efficiency of the PG-based protocol and even achieved a comparable efficiency to the conventional P4-based method. Considering the lower cost and easier operation of the PGP protocol than the P4-eCG protocol, it may be a potential alternative to the conventional P4-eCG based protocol.

## Figures and Tables

**Table 1:** Effect of three estrus synchronization protocols on estrus traits of Mongolian sheep (experiment 1).

Parameters	P4-eCG Group (*n* = 10)	PG Group (*n* = 10)	PG-GnRH-PG Group (*n* = 10)	*p* Value
Estrus rate (%)	100.0 (10/10)	100.0 (10/10)	100.0 (10/10)	n.s.
Interval from sponge removal or second PG injection to estrus onset (h)	44.0 ± 3.5 ^b^	50.4 ± 3.0 ^a^	52.4 ± 2.6 ^a^	*p* = 0.026
Estrus duration (h)	40.0 ± 6.9	43.2 ± 6.0	38.8 ± 6.3	n.s.
Interval from estrus onset to ovulation (h)	25.2 ± 2.9	24.9 ± 3.6	23.1 ± 2.4	n.s.
Interval from sponge removal or second PG injection to ovulation (h)	63.6 ± 2.4 ^b^	71.1 ± 3.6 ^a^	73.1 ± 4.5 ^a^	*p* = 0.037
Diameter of the largest follicle at estrus onset (mm)	6.7 ± 0.3	6.8 ± 0.3	6.2 ± 0.3	n.s.
Diameter of the second largest follicle at estrus onset (mm)	5.7 ± 0.3	5.0 ± 0.6	5.0 ± 0.3	n.s.
Diameter of large preovulatory follicles (mm)	6.9 ± 0.3	7.0 ± 0.3	6.4 ± 0.3	n.s.
Occurrence of ovulation (%)	100.0 (10/10)	100.0 (10/10)	100.0 (10/10)	n.s.

Estrus rate: number of estrous ewes/number of total ewes. Occurrence of ovulation: number of ovulating ewes/number of estrous ewes. P4-eCG group: FGA (45 mg) sponge for 14 days + 330 IU eCG on Day 0. PG group: two doses of PG (0.1 mg) with 14-day interval. PG-GnRH-PG group: two doses of PG (0.1 mg) with 14-day interval + 50 μg GnRH on Day 7. Data for estrus rate and occurrence of ovulation are presented as percentage, remaining data are presented as Mean ± SEM. a and b: values within a rows having different superscripts differ significantly (*p* < 0.05). n.s.: not significant (*p* > 0.05).

**Table 2:** Effect of three estrus synchronization protocols on reproductive traits of Mongolian sheep (experiment 2).

Parameters	P4-eCG Group (*n* = 20)	PG Group (*n* = 20)	PG-GnRH-PG Group (*n* = 19)	*p* Value
Estrus rate (%)	95.0 (19/20)	100.0 (20/20)	100.0 (19/19)	n.s.
Occurrence of ovulation (%)	94.7 (18/19)	100.0 (20/20)	100.0 (19/19)	n.s.
Ovulation rate (*n*)	1.8 ± 0.1 ^a^ (33/18)	1.3 ± 0.1 ^b^ (25/20)	1.4 ± 0.1 ^b^ (27/19)	*p* = 0.004
Pregnancy rate (%)	70.0 (14/20)	60.0 (12/20)	84.2 (16/19)	n.s.
Lambing rate (%)	60.0 (12/20)	60.0 (12/20)	84.2 (16/19)	n.s.
Litter size (*n*)	1.4 ± 0.2 (17/12)	1.2 ± 0.1 (14/12)	1.3 ± 0.1 (21/16)	n.s.
Single lamb rate (%)	58.3 (7/12)	83.3 (10/12)	68.8 (11/16)	n.s.
Twinning rate (%)	41.7 (5/12)	16.7 (2/12)	31.3 (5/16)	n.s.

Estrus rate: number of estrous ewes/number of total ewes. Occurrence of ovulation: number of ovulating ewes/number of estrous ewes. Ovulation rate: number of corpus luteum/number of ovulated ewes. Pregnancy rate: number of pregnant ewes/number of total ewes. Lambing rate: number of lambed ewes/number of total ewes. Litter size: number of total lambs/number of lambed ewes. Single lamb rate: number of ewes lambed single/number of lambed ewes. Twinning rate: number of ewes lambed twins/number of lambed ewes. Female lamb rate: number of female lambs/number of total lambs. Male lamb rate: number of male lambs/number of total lambs. P4-eCG group: FGA (45 mg) sponge for 14 days + 330 IU eCG on Day 0. PG group: two doses of PG (0.1 mg) with 14-day interval. PG-GnRH-PG group: two doses of PG (0.1 mg) with 14-day interval + 50 μg GnRH on Day-7. Data for ovulation rate and litter size are presented as Mean ± SEM; remaining data are presented as percentage. a and b: values within a rows having different superscripts differ significantly (*p* < 0.05). n.s.: not significant (*p* > 0.05).

**Table 3:** Reproductive performance after TAI in Hu sheep synchronized with two estrus synchronization protocols.

Parameters	P4-eCG Group	PG-GnRH-PG Group	*p* Value
Pregnancy rate (%)	63.4 (90/142)	69.9 (100/143)	n.s.
Lambing rate (%)	59.2 (84/142)	66.4 (95/143)	n.s.

Pregnancy rate: number of pregnant ewes/number of total ewes. Lambing rate: number of lambed ewes/number of total ewes. P4-eCG group: FGA (45 mg) sponge for 14 days + 330 IU eCG on Day 0. PG-GnRH-PG group: two doses of PG (0.1 mg) with 14-day interval + 50 μg GnRH on Day 7. Data are presented as percentage. n.s.: not significant (*p* > 0.05).

## Data Availability

The data presented in this study are available on request from the corresponding author.
